# Central Pathophysiology and Brain Network Changes Related to Camptocormia in Parkinson's Disease

**DOI:** 10.1002/mds.30278

**Published:** 2025-06-29

**Authors:** Tauqeer Anjum, Jan Raethjen, Hao Ding, Nabin Koirala, Rüdiger Pryss, Robin Wolke, Chi Wang Ip, Günther Deuschl, Jens Volkmann, Nils G. Margraf, Muthuraman Muthuraman

**Affiliations:** ^1^ Department of Neurology University Hospital Würzburg Germany; ^2^ Department of Neurology Kiel Germany; ^3^ Brain Imaging Research Core (BIRC) University of Connecticut Storrs Connecticut USA; ^4^ Child Study Center, School of Medicine Yale University New Haven USA; ^5^ Center for Biomedical Imaging & Neuromodulation Computational Neuroimaging Laboratories Nathan Kline Institute for Psychiatric Research (NKI) New York USA; ^6^ Institute of Clinical Epidemiology and Biometry University of Würzburg Würzburg Germany; ^7^ Institute of Medical Data Science University Hospital Würzburg Würzburg Germany; ^8^ Informatics for Medical Technology, Institute of Computer Science University of Augsburg Augsburg Germany

**Keywords:** electroencephalography, electromyography

## Abstract

**Background:**

Studies on brain connectivity offer important insights into the changes that occur in central network diseases such as Parkinson's disease (PD). Camptocormia, a condition characterized by abnormal flexion of the trunk, often occurs in advanced PD, but its underlying mechanisms are not yet clear.

**Objective:**

This study aims to investigate the changes in the central motor network, associated with camptocormia.

**Methods:**

High‐density 256‐channel electroencephalography (EEG) and multichannel electromyography (EMG) recordings from the paravertebral lumbar (PVL) muscles were obtained. Tomographic maps of EEG–EMG coherence were obtained using a beamformer algorithm based on individual β frequencies. We employed temporal partial directed coherence analyses to estimate the direction of information flow between coherent sources.

**Results:**

In all patients and healthy controls, we found a widespread central network that showed coherence with PVL muscles and consisted of cortical and subcortical regions mostly with mutual interactions on the central level and with the paravertebral muscles. In camptocormia patients, there was a significantly stronger coherence between the cortical and muscle activity than in PD without camptocormia patients. Brainstem activation was absent in camptocormia patients, whereas in PD without camptocormia patients, coherent activation in the posterior parietal cortex was missing.

**Conclusion:**

Increased central muscle drive to the paravertebral muscles in camptocormia patients may indicate a relatively simple compensatory strategy to overcome stooped posture, whereas the decreased interaction strength between the central coherent sources and the lack of brainstem involvement likely reflects the primary deficits in central postural control network, likely promoting the defective postural motor pattern in camptocormia. © 2025 The Author(s). *Movement Disorders* published by Wiley Periodicals LLC on behalf of International Parkinson and Movement Disorder Society.

## Introduction

The pathophysiology of camptocormia remains poorly understood and appears to involve both central and peripheral processes, including dysfunctions within the central nervous system (CNS) and pathological changes in the musculature. From a central perspective, alterations in brainstem and cortical activation patterns have been reported in affected patients. On the peripheral level, an empirically established monomorphic myopathy of the paravertebral muscles is observed, exhibiting different stages from acute to chronic degeneration. However, the relationship between central dysfunction and muscular pathology remains unclear. This study aims to bridge this gap by investigating the interplay between CNS mechanisms and myopathy in camptocormia.

Camptocormia as an involuntary nonfixed pathological flexion of the trunk in advanced stages of Parkinson's disease (PD) is still an unsolved riddle. Even with improvements in diagnostical standards in the field of axial postural abnormalities, we still lack an empirical‐based pathophysiological understanding of what is going on behind the clinical scenario. This is an obstacle to the development of effective therapeutic strategies.

Approximately 7% of PD patients suffer from camptocormia as a painful, disabling, and immobilizing severe complication^1^, ^2^. It is acknowledged that myopathy of the paravertebral muscles is involved in most of these PD cases, presenting in varying stages from acute to chronic muscle alterations^3^, ^4^, ^5^. However, the etiology of this process is still unknown. Reports on positive effects of deep brain stimulation (DBS) in camptocormia patients hint at a central process being most likely involved^6^, ^7^. The empirically proven monomorphic myopathy of paravertebral muscles is, therefore, most likely to be understood as a secondary myopathy.

Further support for a central‐caused axial postural abnormality comes from a case report^8^ demonstrating that a lenticular lesion was related to this bent spinal stance, indicating the importance of the basal ganglia (BG) in maintaining the upright posture. However, the connection between secondary myopathy and the well‐known central pathology in PD remains unclear.

Electroencephalography (EEG) studies in patients with PD in a standing position have shown specific cortical network changes and changes in the connectivity between cortical areas and the brainstem depending on the postural task^9^, ^10^. One pathoanatomical substrate of these functional changes may be a decreased volume in certain cerebral regions of PD patients, especially in the occipital lobe, temporal lobe and cortical areas, amygdala, and basal forebrain nuclei^11^.

The present study is aimed at finding out changes in the central network connectivity specifically related to and likely contributing to camptocormia in PD patients. We expect to see changes in cortical–subcortical connectivity patterns mediated through the BG and directional brain region–peripheral connections in camptocormia patients. For this purpose, we use high‐resolution EEG recording source analysis in relation to electromyography (EMG) recordings from affected trunk muscles to find active cortical and subcortical regions and analyze the connectivity between these regions.

## Materials and Methods

### Subjects

This study involved 11 PD patients without camptocormia, 11 PD patients without camptocormia (PD campto), and 11 healthy controls (HC). The diagnosis of PD was established according to the Movement Disorders Society (MDS) clinical diagnostic criteria^12^. Camptocormia was defined as a nonfixed marked anterior flexion of the thoracolumbar spine of at least 30°^2^. The study was approved by the local ethics committee in Kiel, and all the patients provided written informed consent before taking part in our study. The patients and HCs were age matched in each group; however, there were more male PD without camptocormia than PD patients with camptocormia and HCs in each group. The HCs and PD patients with camptocormia were age‐ and sex matched. Details of the subjects are provided in Table 1. Dopamine agonists for patients are provided in Table S1.

**TABLE 1:** Demographics and clinical scores of patients and HCs

Parameters	PD patients without camptocormia	PD patients with camptocormia	HCs
N	11	11	11
Male/female	8/3	6/5	6/5
Mean age in years, mean ± SD	79.2 ± 7.7	82.6 ± 7.4	82.3 ± 6.3
PD disease duration (y), mean ± SD	11 ± 4.1	12.7 ± 4	–
LEDD, mean ± SD	878.1 ± 475.69	795.7 ± 313.96	–
UPDRS, part III (points)	20.4	28.7	–
H&Y stage	2.5	3.5	–

Abbreviations: HC, healthy control; PD, Parkinson's disease; SD, standard deviation; LEDD, levodopa equivalent daily dose; UPDRS, Unified Parkinson's Disease Rating Scale; H&Y, Hoehn and Yahr.

### Tasks and Data Acquisition

EEG was recorded for anterior flexion forward. Bipolar surface EMG was recorded from the paravertebral lumbar (PVL) muscles (L2–L4). Patients and HCs were asked to stand in their comfortable positions. Probands had closed eyes. The camptocormia patients stood in their usual posture. All other probands mimicked a forward bended position. The two electrodes were placed 2.5 cm lateral from the spinous processes about 3 cm apart. EEG was recorded with a high‐density 256‐channel recording system (Electrical Geodesics, Inc., (Eugene, Oregon, USA)[EGI], Philips) with Cz as a reference at a sampling rate of 1000 Hz. The EMG was recorded bipolar simultaneously synchronized to the EEG with the physiological input box, with a sampling rate of 1000 Hz. Data were analyzed offline.

### Data Preprocessing

**FIG. 1:**
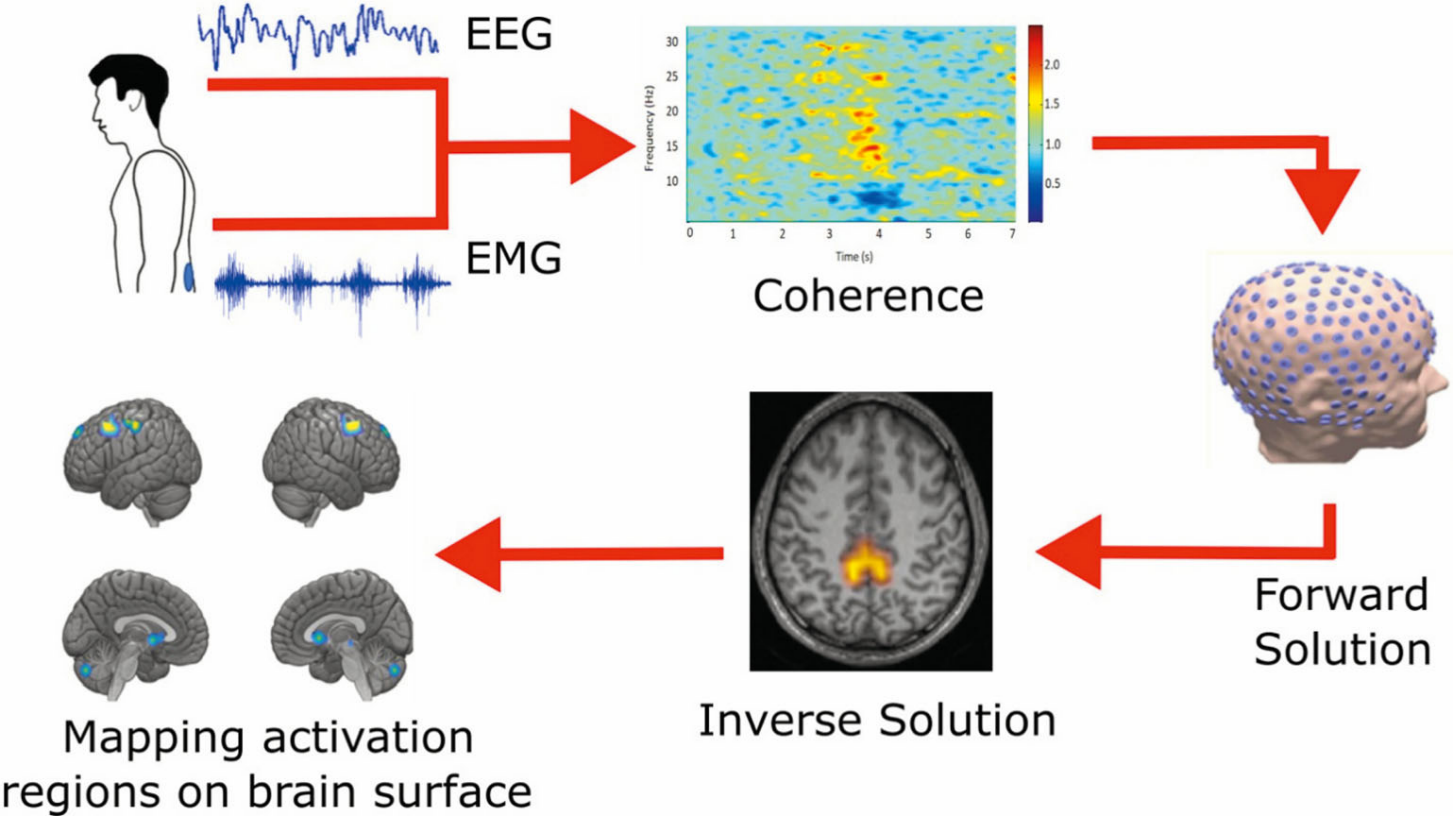
The pipeline used in this study is shown schematically with a representative figure for each step. The raw EEG (electroencephalography) and EMG (electromyography) data after preprocessing (first step) are subjected to time–frequency analyses to estimate the coherence (second step) and to select the time intervals with high coherence. The third step is the forward solution, producing a realistic head model for each subject. The fourth step is the inverse solution to identify the network of sources at a specific frequency. The last step is mapping those source locations in the brain. [Color figure can be viewed at wileyonlinelibrary.com]

The methods used in this study are shown in Figure 1. EEG and EMG were bandpass filtered (EMG: 30–200 Hz, EEG: 0.05–200 Hz). EMG from the PVL (both sides) was full‐wave rectified before filtering, producing demodulated EMG^13^. Each recording was segmented into several 1‐second epochs (L = 1000). All data segments with visible artifacts were discarded. Sixty epochs were eliminated, and no significant differences between groups were found (PD with vs. PD without camptocormia: *P* = 0.348, PD with camptocormia vs. HC: *P* = 0.674, PD without camptocormia vs. HC: *P* = 0.492). Depending on the length (N) of the recording and the quality of the data, 220 to 240 1‐second epochs (M) were used for analysis, such that N = LM.

We used a similar pipeline as in our previous studies,^14^, ^15^, ^16^ starting with coherence and time–frequency analyses followed by source and connectivity analyses. Coherence analysis was performed on the EEG and EMG data using the Welch periodogram method^17^ to estimate the coherence spectrum. Time–frequency analysis was performed using the multitaper method^18^ to assess the dynamics of EEG and EMG signals in both the time and frequency domains. Source analysis was performed to localize brain activity coherent with the peripheral EMG signal by solving the forward and inverse problems (for details see Muthuraman et al.^19^). Finally, the temporal partial directed coherence (TPDC) approach^20^, ^21^ was used for the connectivity analysis, which utilizes dual extended Kalman filtering for dynamic autoregressive coefficient estimation. A detailed description of these methods is presented in the Supplementary Method section.

### Statistical Analysis

The total analyzed data lengths, coherence values, and clinical scores among the three groups of subjects were tested using a nonparametric Friedman test for independent samples (n = 11, *P* = 0.01). The interindividual differences in the source locations within each group of subjects (n = 11, *P* = 0.01) were tested using a nonparametric Kruskal–Wallis test. The statistical significance of the sources (n = 11, *P* = 0.01) was tested using a within‐subject surrogate analysis. A Monte Carlo test of 1000 random permutations was carried out, and the *P*‐values were calculated^22^, ^23^. The *P*‐value for each of these 1000 random permutations was estimated, and then the 99th percentile *P*‐value was taken as the significance level for each subject^24^. A 1000‐ms block length was chosen by an adaptive block length selection method^25^.

## Results

The recorded data lengths (within the subjects) were not significantly different between (each) groups (*P* = 0.651, *P* = 0.553, *P* = 0.234). All identified sources were statistically significant (*P* = 0.002) in a Monte Carlo random permutation test across all subjects within each group. For the common sources, namely prefrontal cortex (PFC), premotor cortex (PMC), leg motor areas (LMA), supplementary motor area (SMA), BG (exact allocation of EEG signal component from deep sources to specific subcortical voxel is difficult), and cerebellum, the peak coherence was at the same location and was not statistically different between the groups of PD patients without camptocormia versus PD patients with camptocormia (*P* > 0.05), PD patients without camptocormia versus HCs (*P* > 0.05), and PD patients with camptocormia versus HCs (*P* > 0.05).

### Clinical Scores

The groups of PD patients with and without camptocormia were compared for differences in their clinical characteristics. There were no significant differences in the disease duration, levodopa equivalent dose, and Unified Parkinson's Disease Rating Scale (UPDRS), part III, scores. Only the H&Y (Hoehn and Yahr) stages were significantly higher (*P* < 0.05) in camptocormia patients, which is not surprising as axial and postural symptoms are stage‐defining symptoms on the H&Y scale (Fig. S1).

### Clinical Correlations

For PD patients with and without camptocormia, the coherence between PVL muscles and the LMA is negatively correlated with levodopa intake (in mg/day). The UPDRS scores are correlated with the cerebral source and PVL muscles in PD patients with camptocormia. In PD patients without camptocormia, there was a positive correlation with the UPDRS score for the coherence between the brainstem source and the PVL muscle.

TPDC reveals a causal connection from the BG to the cerebellum in PD patients with camptocormia. For PD patients without camptocormia, there is a causal connection from the cerebellum to the brainstem. The strengths of those connections plotted against the UPDRS score (Fig. S2) show a downward trend with the increase in UPDRS score. No other significant correlations between clinical characteristics and the connectivity measures between the muscle and brain areas or within the central network were found.

### The Central Coherent Network Related to Postural Muscle Activity

Time intervals having significant coherence between EMG and EEG were found for all three groups, and the mean coherence values were 0.28 for PD patients with camptocormia, 0.21 for PD patients without camptocormia, and 0.14 for HCs at the β frequency. The mean frequency of the coherence peaks in the β range was 17.72 Hz for PD patients with camptocormia, 16.36 Hz for PD patients without camptocormia, and 25.36 Hz for HCs. The lower β‐frequency coherence observed in the PD patient groups with and without camptocormia may reflect impaired motor function and reduced network efficiency, consistent with the findings from our previous study^26^.

In HCs, the source signals from the region of the LMA, PMC, SMA, medial prefrontal cortex (MPFC), BG (likely thalamus), posterior parietal cortex (PPC), brainstem, and cerebellum exhibited significant coherence with the surface EMG from the PVL muscles.

**FIG. 2:**
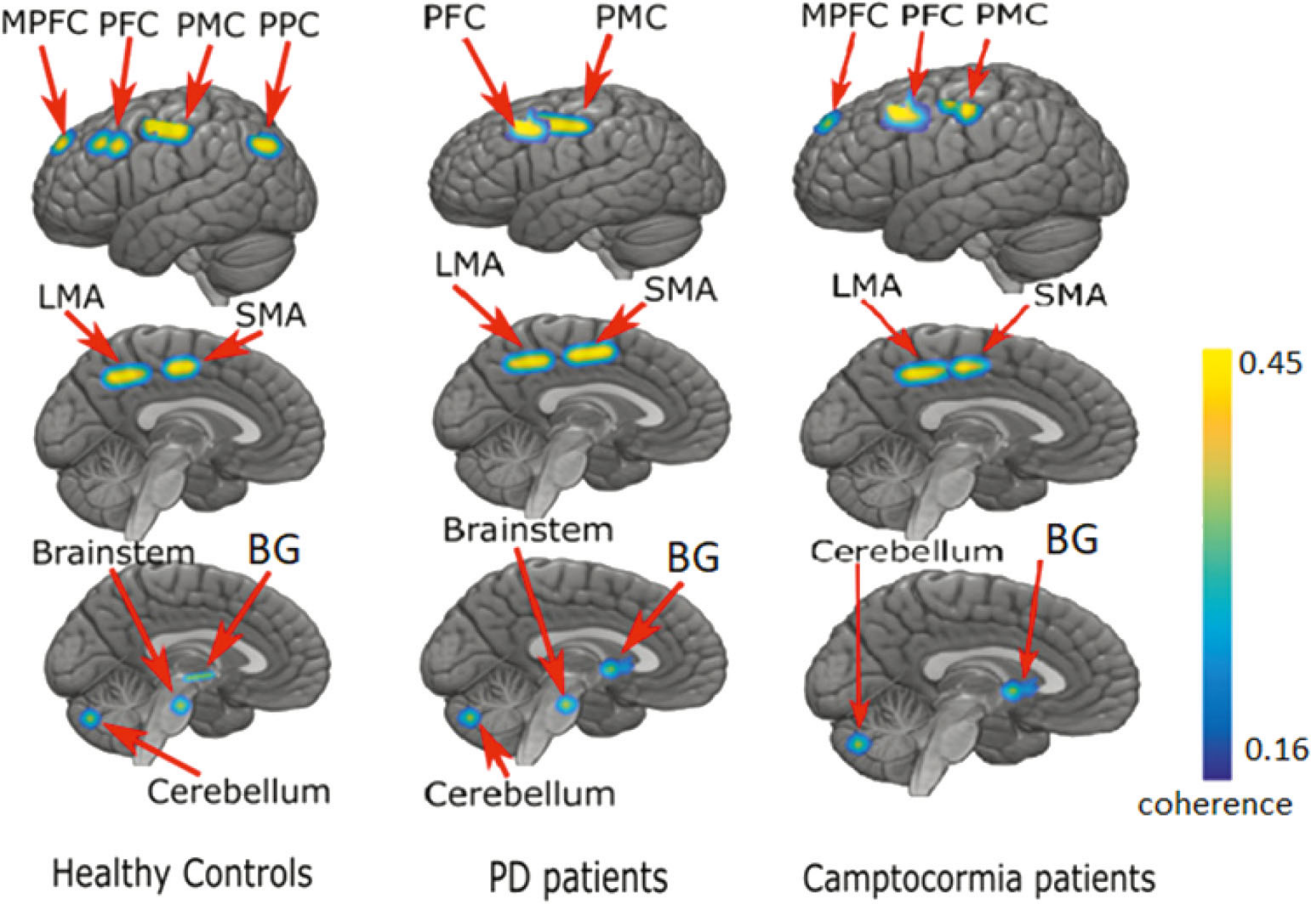
This figure shows the activation in different regions of the brain for a group of healthy controls (left), Parkinson's disease (PD) patients without camptocormia (center), and PD patients with camptocormia (right). Nine areas in total show brain activation, namely the premotor cortex (PMC), prefrontal cortex (PFC), medial prefrontal cortex (MPFC), posterior parietal cortex (PPC), supplementary motor area (SMA), leg motor area (LMA), basal ganglia (BG), cerebellum‐CERE‐VIIa lobule (cerebellum‐CE), and brainstem pons (brainstem). [Color figure can be viewed at wileyonlinelibrary.com]

In all the patients, a similar but less widely distributed central network showed coherence with the PVL muscle signal. Specifically in PD patients without camptocormia, the PPC and MPFC did not show PVL muscle‐related coherent activity. In PD without camptocormia patients, not only was the PPC missing in the network, but also the brainstem region did not show coherent activity. The details of the coherent regions for HCs, PD without camptocormia patients, and PD with camptocormia patients are shown in Figure 2.

### Directions of Interactions in the Network

#### Interactions with the Central Network

In the HC group, there are causal connections within cortical and subcortical regions from the PMC to the PFC and SMA, the SMA to the PFC, and the PFC to the MPFC and BG. The BG has a connection to the LMA and PPC. In the subcortical structure, the brainstem has a connection to the BG and the cerebellum.

In the group of PD patients without camptocormia, there were causal connections within the cortical regions, from the LMA to the PMC, and from the PMC to the SMA and PFC. The cortical and subcortical regions are connected from the PFC to the BG (likely caudate) and then from the BG to the LMA and SMA. In the subcortical structure, the cerebellum shows connectivity to the BG and the brainstem.

**FIG. 3:**
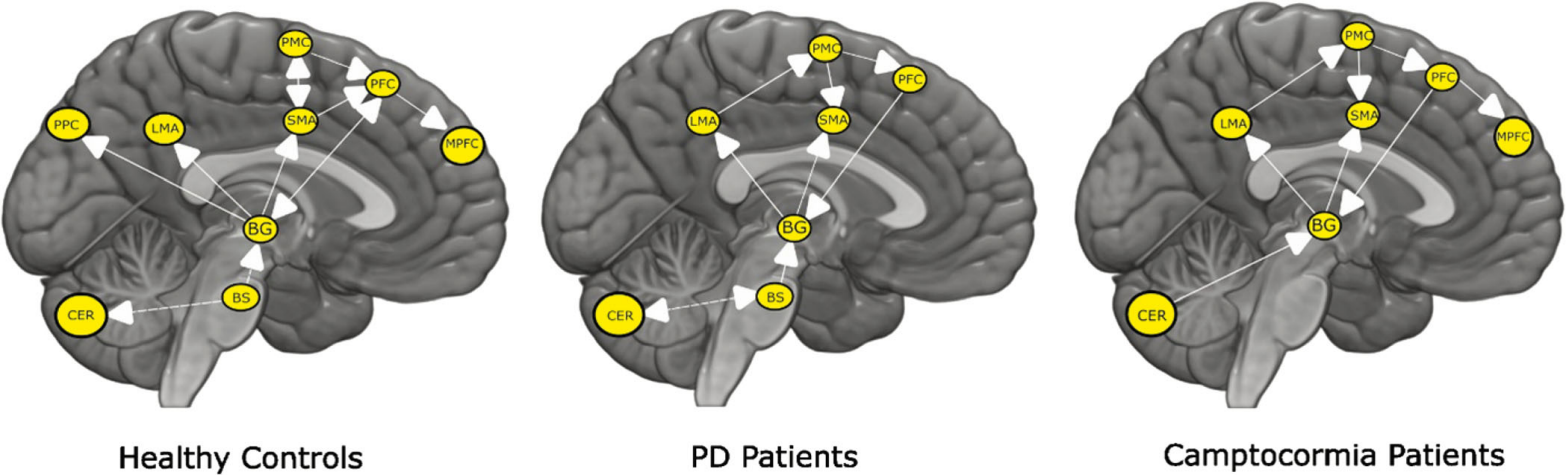
The figure contains a set of three brain images showing unidirectional/bidirectional connections within different activation areas of the brain for a set of healthy controls (left), Parkinson's disease (PD) patients without camptocormia (center), and PD patients with camptocormia (right). The activation regions found above are used here for the respective groups. These regions are the leg motor area (LMA), premotor cortex (PMC), supplementary motor area (SMA), prefrontal cortex (PFC), medial prefrontal cortex (MPFC), posterior parietal cortex (PPC), basal ganglia (BG), cerebellum‐CERE‐VIIA lobule (CER), and brainstem pons (BS [basal ganglia]). [Color figure can be viewed at wileyonlinelibrary.com]

In camptocormia patients, there are causal connections within the cortical regions from the LMA to the PMC, the PMC to the SMA, and the PFC. The PFC is connected to the MPFC and subcortical regions through the BG. BG is also connected to the cortical regions such as the LMA and SMA. The subcortical structures are connected from the cerebellum to the BG (likely putamen). The brainstem is a missing coherent region in PD patients with camptocormia; therefore, there is no connection to the brainstem. The details of the connections for all three groups are shown in Figure 3.

#### Interactions between Central Coherent Sources and Postural Muscle Activity

In the HCs, there are connections from both the cortical and subcortical regions such as the LMA, SMA, PFC, and brainstem to the periphery. We also observed connectivity from the periphery to the subcortical regions like the BG and cerebellum and cortical regions like the PMC and MPFC.

In PD patients without camptocormia, there are causal connections from the LMA, PMC, BG, and brainstem to the periphery. We observe a connection from the periphery to the cortical regions such as the SMA and PFC, and subcortical region such as the cerebellum.

**FIG. 4:**
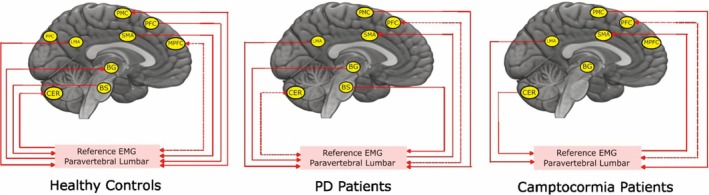
The figure contains a set of three brain images showing unidirectional/bidirectional connections from different activation areas of the brain to the paravertebral lumbar (PVL) muscles in the periphery for a set of healthy controls (left), PD patients without camptocormia (center), and PD patients with camptocormia (right). The activation regions found above are used here for the respective groups. These regions are the leg motor area (LMA), premotor cortex (PMC), supplementary motor area (SMA), prefrontal cortex (PFC), medial prefrontal cortex (MPFC), posterior parietal cortex (PPC), basal ganglia (BG), cerebellum‐CERE‐VIIA lobule (CER), and brainstem pons (BS). [Color figure can be viewed at wileyonlinelibrary.com]

The camptocormia patients also show a connection from the LMA, PMC, PFC, and cerebellum to the periphery. We observed connections from the periphery to only the cortical regions of the brain such as the SMA and PFC. The details of the connections are shown in Figure 4.

## Discussion

Previous studies have shown that DBS of the subthalamic nucleus or globus pallidus internus can be effective in treating camptocormia.^6^, ^7^ These results indicate that postural abnormality like camptocormia in PD might be a disorder with central etiology. Our study focuses on the brain structures and the involved networks in axial postural abnormality using EMG as a reference. We studied the involved network similarities and differences between a set of HCs, PD patients without camptocormia, and PD patients with camptocormia. The interaction strengths within the central EMG‐related network and between the subcortical or cortical sources and the paravertebral EMG exhibited clear correlations with the UPDRS and levodopa equivalent dose, indicating its connection with the clinical parkinsonian syndrome.

Using high‐density EEG, we found different coherent sources in PD patients with and without camptocormia and HCs. We found that there are mostly similar coherent sources for all three groups (eg, the leg/trunk motor area, PMC, SMA, PFC, and a cerebellar region), indicating that a relatively widespread physiological motor network is related to keeping up an upright posture in all patients and HCs^27^. However, in addition, the HCs have the MPFC, PPC, and brainstem as coherent regions. PD patients without camptocormia have the brainstem, and PD patients with camptocormia have the MPFC as the additional coherent brain regions. In HCs, MPFC activation may support normal postural adjustments and voluntary trunk stabilization, and in the PD patients with camptocormia, the presence of MPFC activation could suggest a compensatory mechanism attempting to counteract the postural dysfunction.

We found that the PPC is the missing coherent source for both patient groups; thus, it cannot be an important factor in PD camptocormia disease. The PPC is thought to be used for the orientation of the body according to gait studies^28^. Thus, one may speculate that this lack of posterior parietal involvement in postural control may facilitate the development of camptocormia in all PD patients by a weakening of the network.

A striking difference between patients with camptocormia and those without camptocormia was the missing coherent source in the brainstem in camptocormia patients. The brainstem is an important node for movement selection and modulation receiving input from the BG and other motor centers^29^. Brainstem is one of the earliest vulnerable structures in multiple neurodegenerative diseases such as multiple system atrophy and PD, as shown in magnetic resonance imaging studies^30^. Studies show that the brainstem's involvement in postural control and motor regulation can lead to its potential contribution to the development of camptocormia^31^. PD with camptocormia may represent a selective form of PD in which a specific neuronal dysfunction possibly occurs within the brainstem^32^.

There are multiple coherent networks identified in our study that contribute to dystonia, including the cerebellum and BG^33^. A part of the activity in PD patients with and without camptocormia is probably related to axial rigidity and bradykinesia. The muscles involved in camptocormia are abdominal and paravertebral or both^34^. The dystonic activity leading to camptocormia is centered mainly in the trunk flexors like the abdominal muscles, for which we did not conduct the surface EMG recordings. To be confident about the dystonic activity, abdominal muscles need to be recorded in the future studies.

The most consistent finding differentiating PD patients with camptocormia from PD patients without camptocormia was in the coherence values between the different coherent sources and the postural muscle signal. For all cortical sources and the cerebellar source, we found a similar pattern with a weaker connection for the PD patients compared to the HCs and a connection strength that was similar to the HCs for the PD patients with camptocormia. At first glance, it seems a paradoxical finding that patients with an additional disabling symptom show a more normal connection strength than patients without this postural disturbance and, likely, this difference between PD with camptocormia and PD without camptocormia patients does not reflect an increase in functional disturbance of the central–peripheral postural motor network. It could rather indicate a compensatory increase in the central drive to the postural muscles, possibly as a reaction to a postural control deficit (eg, due to the lack of posterior parietal involvement) being the initial reason for the stooped posture in these patients.

For the main motor output from the primary motor area of the postural muscles, the cortico‐muscular coherence values were not only significantly stronger for PD patients without camptocormia but even stronger for HCs. The lack of involvement of the brainstem as an important central processing hub^35^ only in camptocormia patients does not only show a deficit in the central network but may also indicate that the output from the postural motor network relies more on the (pre)motor cortex, whereas in PD patients without camptocormia postural control through subcortical output is more common and also indicated by the coherence between the BG source and postural muscles, which were stronger in PD patients without camptocormia than in camptocormia patients and HCs^31^, ^36^.

How could these changes be related to the vast evidence of myopathic changes in postural muscles in camptocormia patients^3^, ^4^? As this myopathy is typically limited to the paravertebral muscles that keep up the erect posture, it has been argued that it could be a secondary phenomenon. The postulated compensatory increase in cortical drive to the paravertebral postural muscles shown here may be the starting point for a vicious circle finally leading to a secondary myopathy, initially compensating for the deficit in controlling an upright posture but putting too much strain on the paravertebral muscles, which then contributes to the damage of paravertebral muscle tissue we see in advanced camptocormia^37^.

Such a gradual emergence of camptocormia in PD with sequential pathophysiological mechanisms could explain why dopaminergic agents can be effective against camptocormia^38^ in some patients possibly before the emergence of irreversible muscle damage. Our present finding of in‐part compensatory changes in the central motor network related to the affected paravertebral muscles and being specific to camptocormia patients may be one explanation for the efficacy of network modulation by DBS. It likely indicates that the postulated vicious cycle of a compensatory increase in postural motor network output and muscle damage is still active and can be interrupted. However, intervention with DBS can possibly be effective only before the effector organ of the PVL muscles is irreversibly damaged by a too long‐lasting abnormal trunk posture.

Within the central postural motor network, the cortical–subcortical connections were reduced in the camptocormia patients compared to the PD patients without camptocormia. Thus, in contrast to the possibly compensatory increase in the cortical drive to the postural muscles, the information flow between the different levels of the central network appears to be decreased in camptocormia, also reflected by the lack of brainstem involvement in camptocormia patients. This could suggest additional computational deficits in the central motor network of camptocormia patients possibly impeding central compensatory strategies and shifting compensation to the output level in the shape of an increased drive to the postural muscles. Due to the vast connections between the cerebellum, BG, and cortical regions, both receiving input from and transmitting output to the various cortical areas in learning and control processes^39^, ^40^ these interactions are likely to play a crucial role also in postural control.

The idea of an increased drive to the muscle as a compensatory mechanism that leads to the structural changes and weakness is purely speculative. It is one of many possible explanations of higher observed coherence in the PD patients with camptocormia. A possible explanation to this increased drive can be a pathological central mechanism with reduced inhibitory control of the motor circuits. This reduced control could also have a similar effect on the muscles. Another explanation, although unlikely, can be purely peripheral, such as changes in the surface EMG spectrum of myopathic muscles, leading to increased coherence. A reason for that is that we observed marked differences in coherence between PD patients without camptocormia and HCs, and neither group exhibited peripheral muscle changes at the time of recordings.

The study indicates alterations in connectivity patterns between subcortical regions, particularly the cerebellum, and peripheral muscles in camptocormia patients. The findings suggest a complex interplay between brain regions and muscles; specifically, healthy individuals exhibit robust connections between peripheral muscles and the cerebellum, indicating efficient sensorimotor integration and feedback mechanisms^41^. Furthermore, our study is the first to provide direct empirical evidence of the central etiology of camptocormia and establishes a link between the already‐known consistent pathological findings in the paravertebral muscles and central postural control.

## Limitations

This study has some limitations related to the cohort of patients. The PD patients without camptocormia is a male‐dominant group. Although sample size estimation was performed (Fig S9.), the sample size might not be sufficient, and further studies with a large sample size of PD patients with camptocormia are required to examine the factors affecting their brain disconnections causing postural abnormalities. The brainstem cluster observed in the control groups might reflect a different motor program rather than the pathological mechanisms underlying camptocormia, which needs to be interpreted with caution. Furthermore, the voluntary effort by controls to hold the stooped posture might activate a central motor network that does not play a role in the emergence of pathological camptocormia. The volumetric beamforming may be suboptimal for accurately localizing lower signal‐to‐noise ratio cortical sources. Future work can address this by incorporating a hybrid approach of surface‐constrained modeling for cortical and volumetric modeling for subcortical sources.

## Conclusions

We can conclude that the brainstem and PPC are important regions to investigate in PD patients with camptocormia and that there is an increased central muscle drive to the PVL muscles in PD patients with camptocormia, indicating a compensatory mechanism to overcome the postural abnormalities. Neuroimaging methods with increasing spatial and temporal resolution will be able to identify supplementary differences in Parkinson's disorders, elevating our understanding of disease pathophysiology.

## Author Roles

1. Research project: A. Conception, B. Organization, C. Execution.

2. Statistical analysis: A. Design, B. Execution, C. Review and critique.

3. Manuscript preparation: A. Writing of the first draft, B. Review and critique.

T.A.: 1A, 1B, 1C, 2A, 2B, 3A

J.R.: 1B, 1C, 3B

H.D.: 2A, 2C

N.K.: 1B, 3B

R.P.: 2C, 3B

R.W.: 2C, 3B

I.C.W.: 1B, 3B

G.D.: 1B, 3B

J.V.: 1B, 3B

N.G.M.: 1A, 1B, 1C, 2A, 2B, 3B

M.M.: 1A, 1B, 1C, 2A, 2B, 3B

## Full financial disclosures of all authors for the past 12 months

The authors declare that there are no conflicts of interest relevant to this work. This study was funded by the Deutsche Forschungsgemeinschaft (DFG, German Research Foundation), project‐ID 424778381–TRR 295.

## Supporting information


**Data S1.** Supporting Information.

## Data Availability

The data that support the findings of this study are available on request from the corresponding author. The data are not publicly available due to privacy or ethical restrictions.
