# Supplementary material for: Central Pathophysiology and Brain Network Changes Related to Camptocormia in Parkinson's Disease
Source: Mov Disord. 2025 Jun 29;40(10):2149–57. doi: 10.1002/mds.30278 (PMC12553991; doi:10.1002/mds.30278)
Supplement: Supplementary file 1 — Data S1. Supporting Information. [file MDS-40-2149-s001.docx]

**Supplementary Methods**

**Coherence analysis**

The coherence spectrum was estimated for the collected high resolution 256 channel EEG and EMG (PVL-muscle) data set, using the Welch periodogram method^1^. The statistical significance^2^ of the coherence at a particular frequency is calculated by:

$1-\left( 1-\chi\right)^{\frac{1}{M-1}}$ eq. 1

Where $\chi$ is set to 0.99, so that the confidence limit is $1-\left( 1-0.01 \right)^{\frac{1}{M-1}}$ . Values of coherence above this confidence limit are considered to indicate statistically significant linear correlation between the two-time series. On the other hand, values below this limit indicate the absence of correlation. In our next step we analysed the dynamics of these frequency oscillations over time by applying the multitaper method for each recording^3^.

**Time-Frequency Analysis**

To compute the dynamics of signals in the frequency and time domains for high resolution 256 channel EEG and EMG (paravertebral (PVL)-muscle) data, the multitaper method was used^4^. This method uses the K windows (tapers) and the data x(t) multiplication to estimate the spectrum. The complete description of the method can be found elsewhere^3^. Time and frequency resolutions were approximately 50 ms and 1 Hz respectively, with the time step of 50 ms having overlapping windows of 1000 ms.

In a further analysis, a pooled coherence estimate was calculated using all the initial coherence estimates of the individual EEG electrodes. This can be done by computing the individual second-order spectra using a weighting scheme and estimating the coherence to obtain the pooled estimate of the individual EEG electrodes^5^,^6^.

Time intervals having significant coherence between EMG and EEG at the beta frequency were selected for all healthy controls and patients from the pooled time-frequency spectrum.

**Source Analysis**

Brain activity coherent with the peripheral EMG signal can be localized using the brain imaging of the coherent sources^7^. Forward and inverse problems are to be solved if we need to locate the origin of a specific EEG activity on the scalp.

The computation of scalp potentials for a set of neural current sources is known as the forward problem, which can be solved by estimating the lead field matrix with specified models for the brain using a finite-element method^8^. For the forward modelling, the compartment surfaces like the brain, skull, and scalp were extracted from the standard T1 MRI, and the individual electrode locations obtained from a Polhemus system were used.

The inverse problem is finding the relation between the underlying neural activities and the electric potentials recorded on the scalp. The spatial filter can be used to solve this problem by a simple linear transformation^9^. The spatial filter allows signals within a certain frequency band from a particular brain location and attenuates the signals from other locations. The detailed explanation of the forward and inverse solutions is provided elsewhere^10^,^11^.

Dynamic imaging of coherent sources is a beamforming technique^12^,^13^ that uses a spatial filter^9^ to compute tomographic maps of cerebro-muscular coherence in the frequency band of interest. The spatial filter was applied to numerous voxels in these regions, providing each voxel with a distinct value of coherence to a particular reference signal. A voxel size of 2 mm was used in this study.

The strongest beta frequency coherence to the EMG signal was identified in the source in the regions of interest. Because the coherence between the detected regions and themselves is always 1, other coherent areas were identified while this region was projected out of the coherence matrix^14^. Once coherent cerebral cortical and cerebellar areas were identified, their activity was extracted by the spatial filter^9^.

Using statistical parametric mapping (SPM12), the individual maps of the strongest cerebro-muscular coherence were spatially normalized, averaged, and shown on a typical Montreal Neurological Institute (MNI) template brain. To account for the unique anatomical variances between the participants, we additionally built a grid for each one of them in a manner that aligns it with the MNI space. The locations with the highest coherence to the EMG signal are represented as local maxima in the resulting maps.

**Connectivity Analysis**

We can concentrate on a specific frequency and examine the time dynamics of the causality at that frequency using time-frequency causality. Dual-extended Kalman filtering serves as a base for the time-frequency causality estimation approach of temporal partial directed coherence TPDC^15^,^16^. This TPDC method allows time-dependent auto-regressive coefficients to be estimated. The states are estimated by one extended Kalman filter, which then feeds this information to another extended Kalman filter, which then estimates the model parameters and feeds this information back to the first. We calculated the states and model parameters of the system at each time instant using two Kalman filters operating in parallel.

The calculation of causality between the time series employed time-dependent multivariate coefficients. We determined partial directed coherence at each time point by calculating the time-dependent multivariate autoregressive coefficients at each time point. The beta frequency band was used which are the frequencies of the pathological and mimicked standing posture. After estimating the TPDC values, the significance level was calculated from the applied data using a bootstrapping method^17^.

To construct a new time series, we, in essence, split the previous time series into smaller, non-overlapping windows and randomly shuffled the order of these windows. The shuffled time series were fitted with a multivariate autoregressive model, and TPDC was calculated. The shuffling operation was repeated 1000 times, and the average TPDC value was used as the threshold for significance for all connections. This process was performed separately for each patient. The resulting value served as the significance threshold for all connections.

The open-source MATLAB package autoregressive fit (ARFIT) was used for estimating the autoregressive coefficients from the spatially filtered source signals^18^,^19^. We applied the time reversal technique^20^ as a second significance test on the connections already identified by TPDC using a data-driven bootstrapping surrogate significance test.

**Supplemetary Figures**


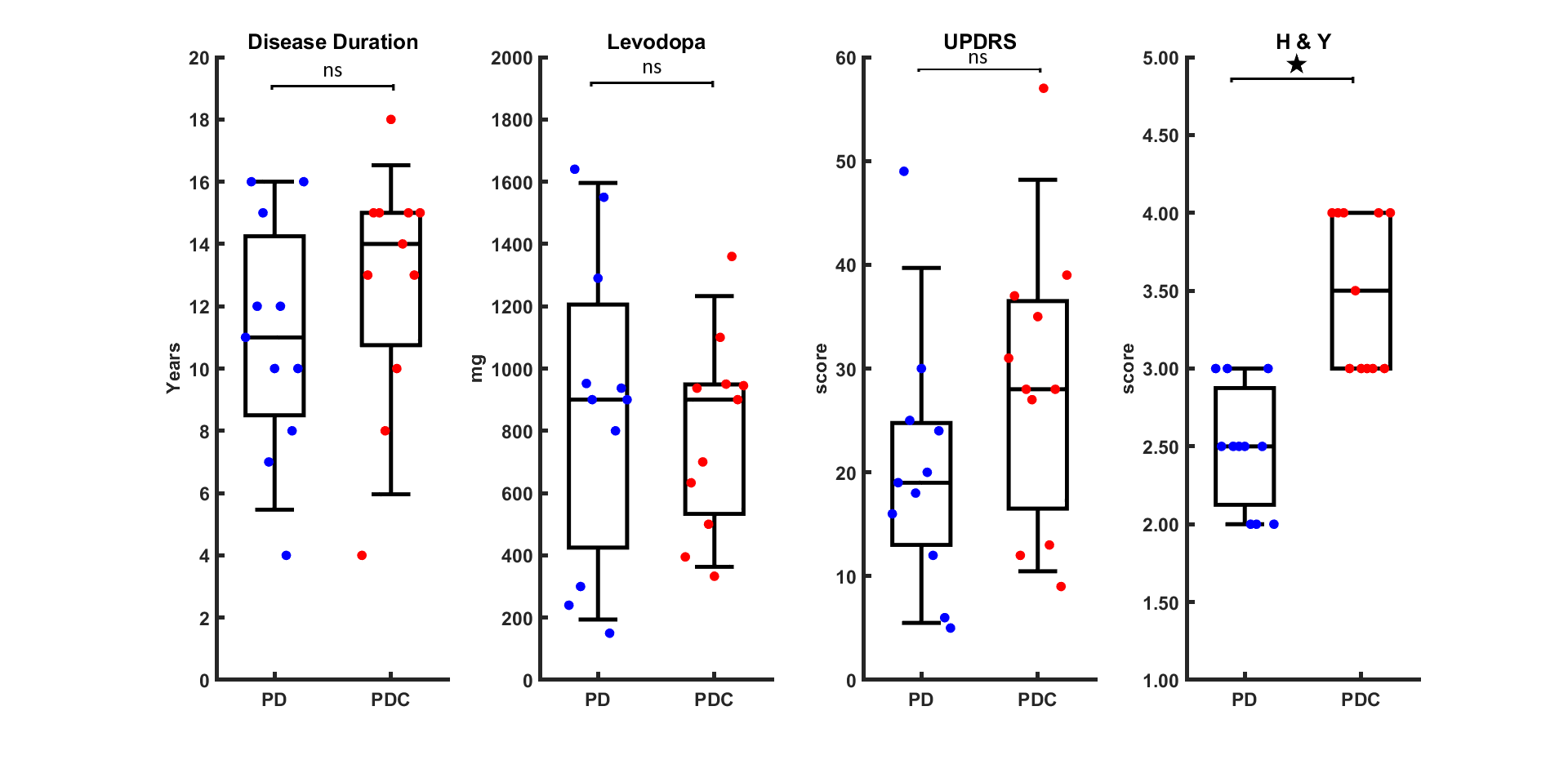


Figure S1: This figure shows the disease duration(left), levodopa-equivalent dose (2^nd^ from left), UPDRS-III scores (3^rd^ from left) and Hoehn and Yahr stage(right) for PD patients without camptocormia (PD) and PD patients with camptocormia (PDC), There are significant differences between the PD and PDC patients in H&Y stages which are marked with a star. No other significant differences were found between the two groups.


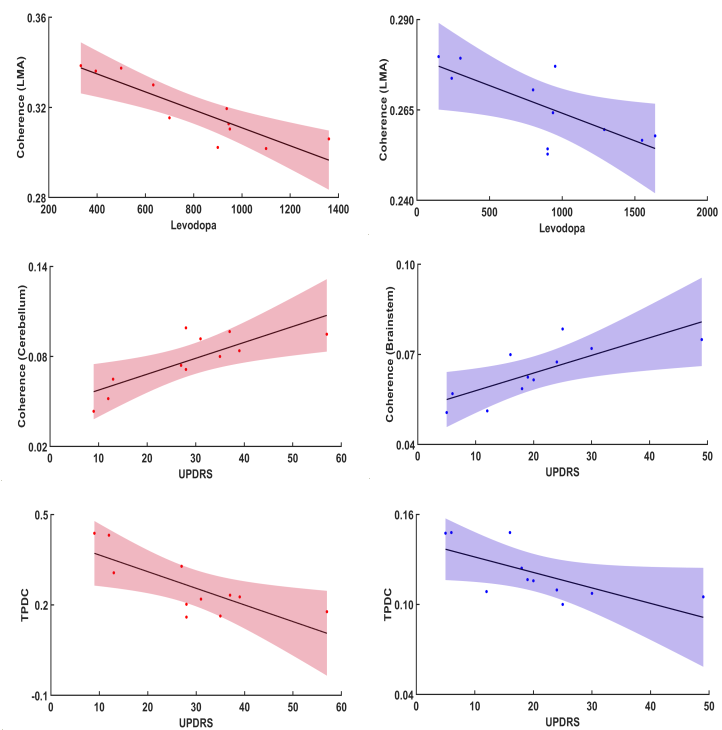


Figure S2: The figure shows the relationship between the coherence values of PD camptocormia patients (red) and PD without camptocormia (purple) and the clinical scores. The curved areas represent the 95% confidence interval. The top row shows the effect of Levodopa intake (in mg/d) and coherence in the LMA coherent source (PD with camptocormia (p=0.0003, R^2^=0.78), PD without camptocormia (p=0.01, R^2^=0.53)). (Centre) Plots show the relationship between coherence values and the UPDRS III scores (Cerebellum for PD camptocormia patients (p=0.003, R^2^=0.64) and Brainstem for PD patients without camptocormia (p=0.0057, R^2^=0.59). Coherence tends to increase with the increasing UPDRS stage III scores. The (bottom) row shows the relationship between TPDC values and UPDRS stage III scores, where TPDC values for PD patients with camptocormia (p=0.0059, R^2^=0.587) and PD patients without camptocormia (p=0.020, R^2^=0.47) tend to decrease with increased UPDRS III scores.

**
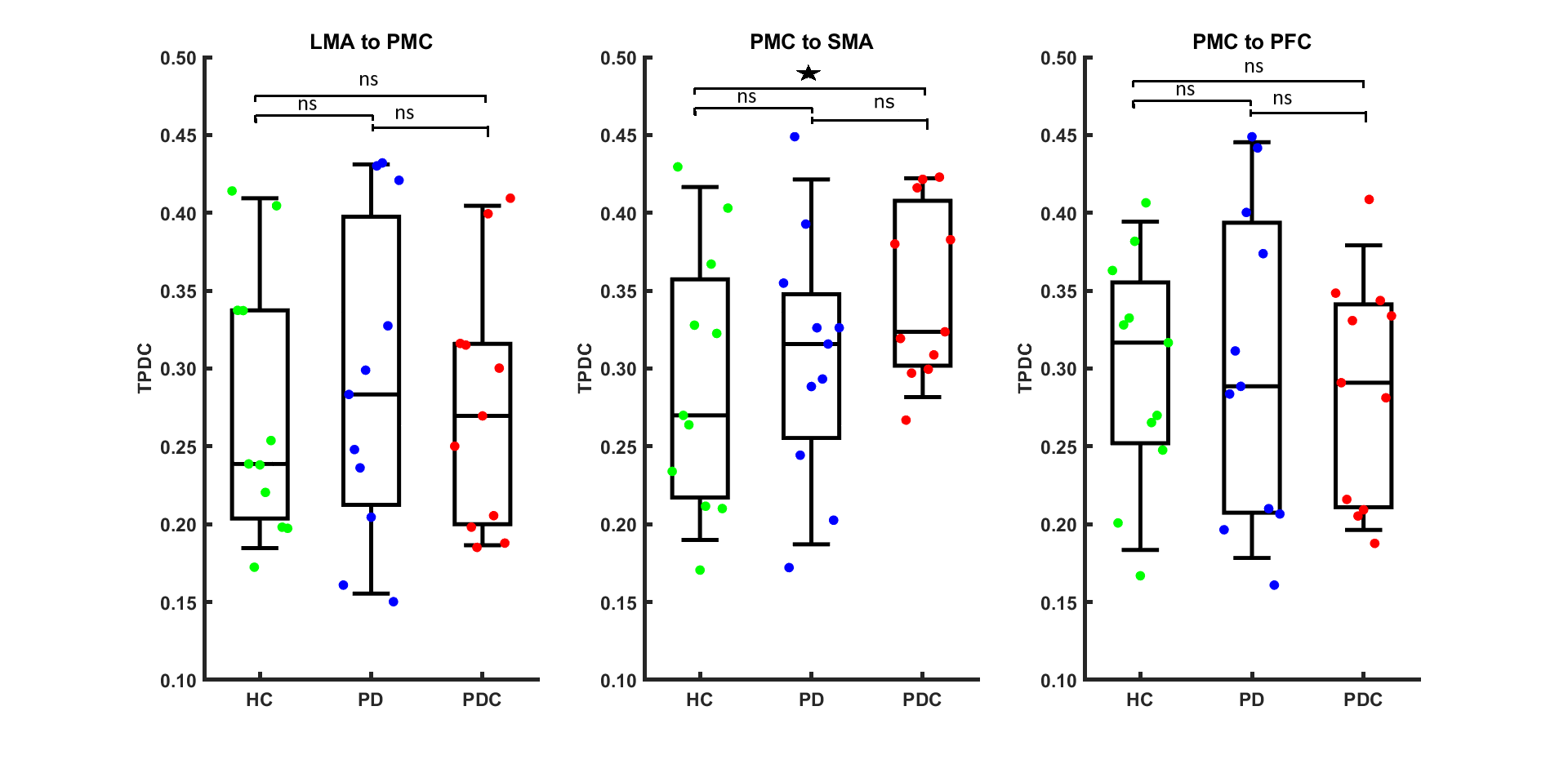
**

Figure S3: This figure shows the temporal partial directed coherence values for common connections for a group of healthy subjects, Parkinson's disease(PD) patients without camptocormia, and PD patients with camptocormia (PDC).

**
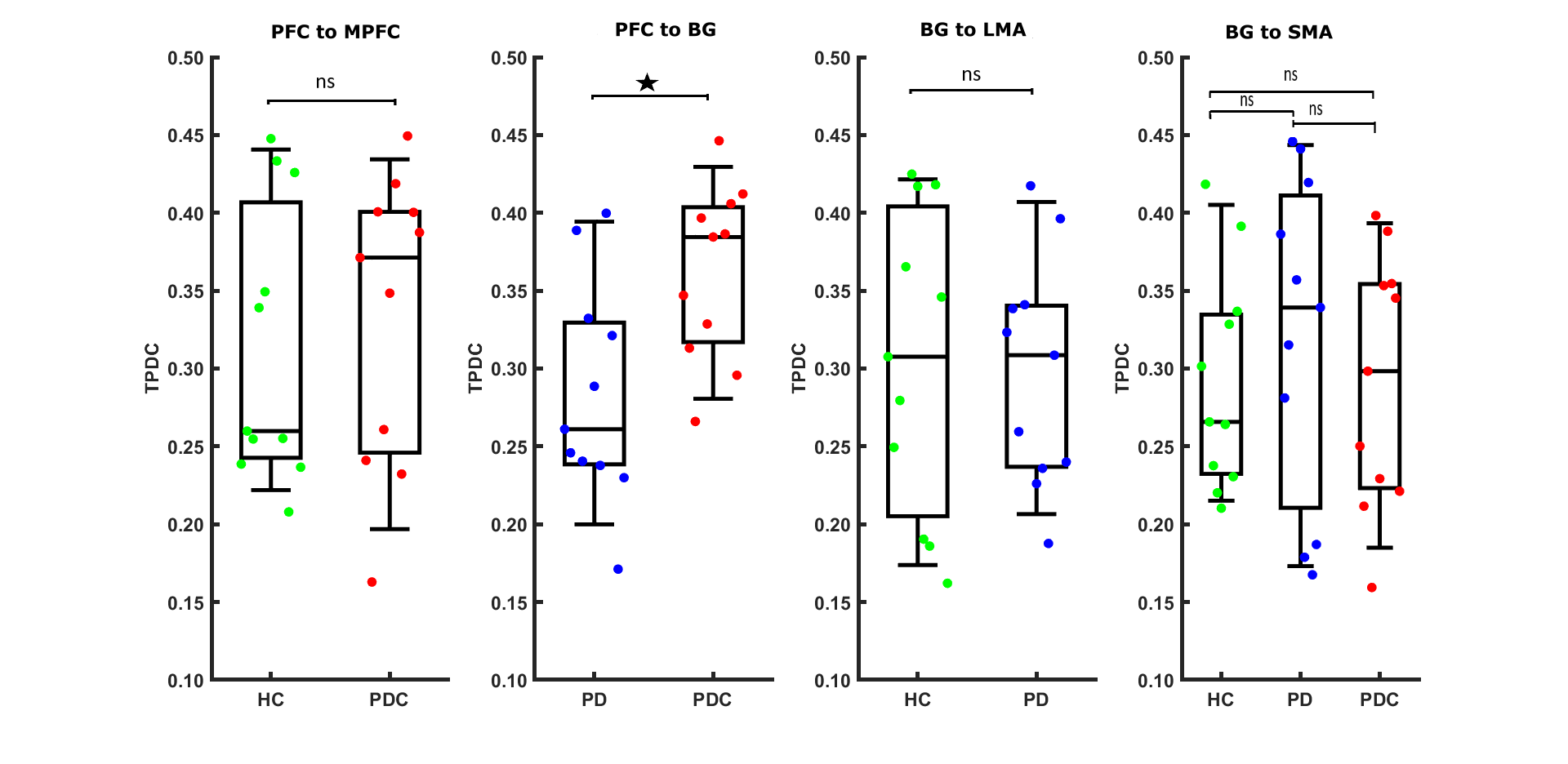
**

Figure S4: This figure shows the temporal partial directed coherence values for common connections for a group of healthy subjects, PD patients without camptocormia (PD), and PD patients with camptocormia (PDC). The connection from PFC to MPFC was found in two groups (HC and PDC), and the connection from PFC to BG was found in both patient groups. The connection from BG to LMA was found in HC and the patient groups. The connection from BG to SMA was found in all the groups.


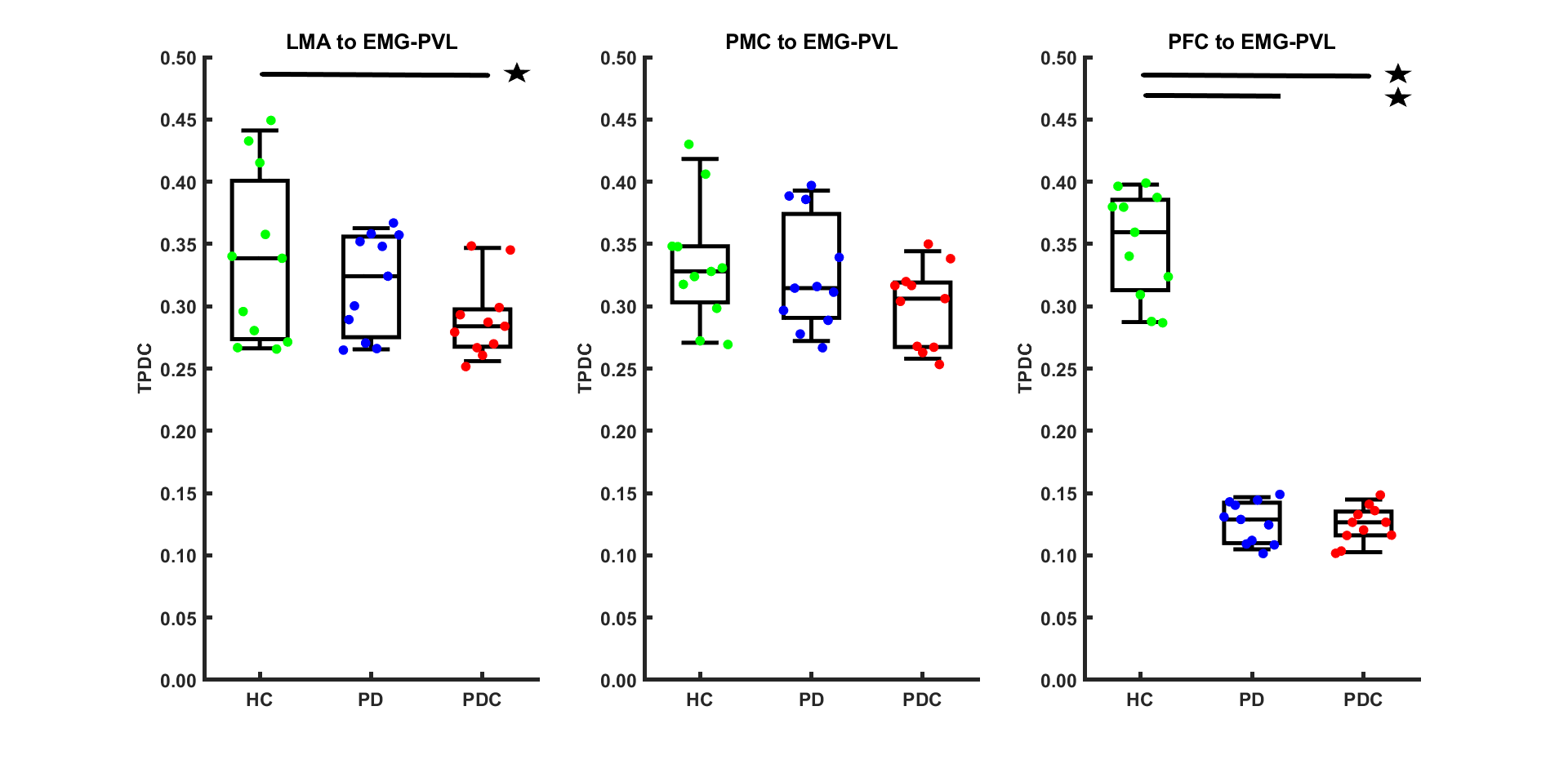


Figure S5: This figure shows the temporal partial directed coherence values for common connections for a group of healthy subjects (HC), PD and PDC patients. The connection from LMA to peripheral muscles, significant difference was found between HC and PDC patients, and for the connection from PFC to peripheral muscles significant differences were found between HC and PD patients as well as HC and PDC patients. No other significant differences were found in the other connections above.


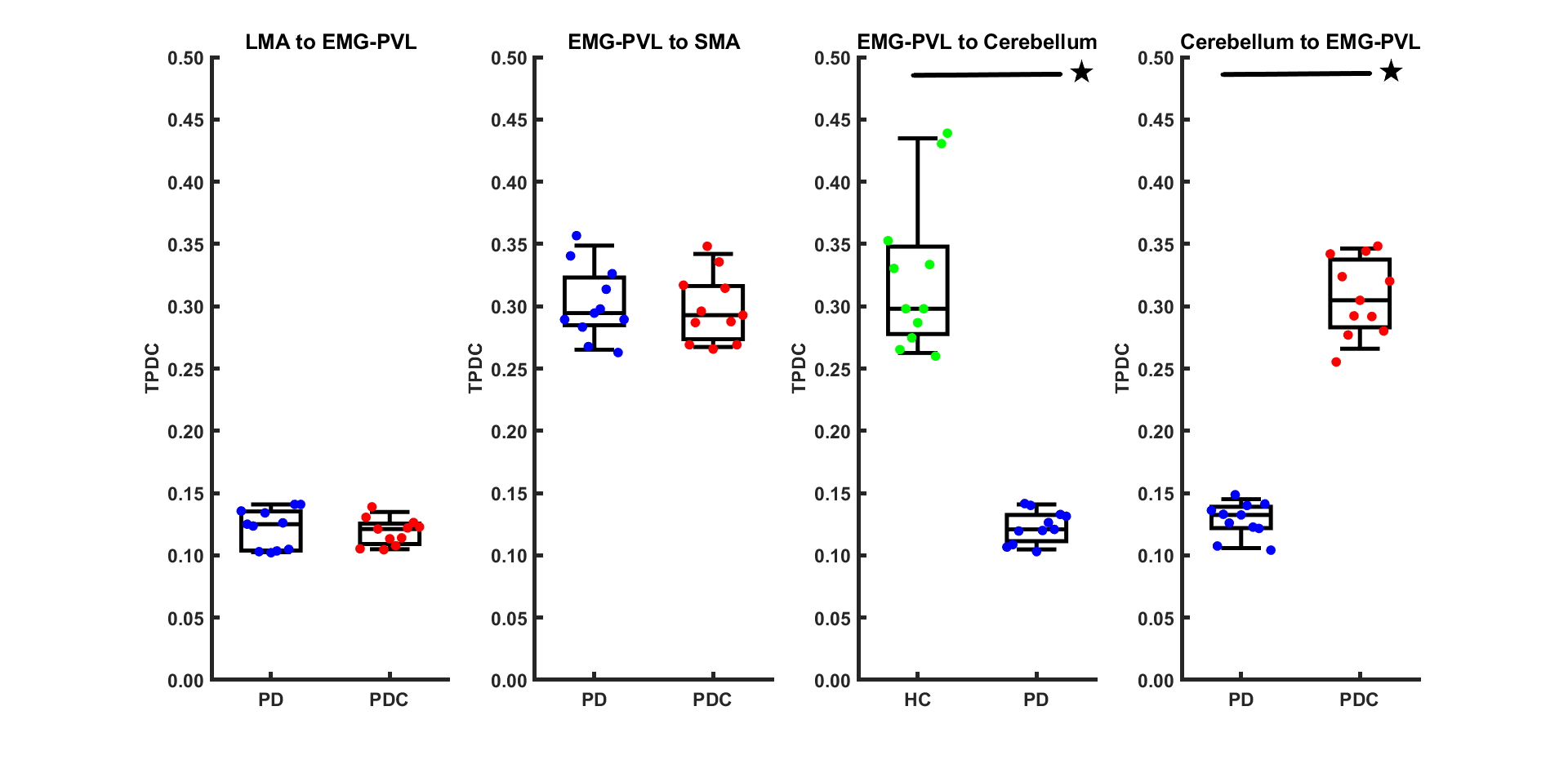


Figure S6: This figure shows the temporal partial directed coherence values for common connections for a group of healthy subjects (HC), PD and PDC patients. The connection from the peripheral muscles to the cerebellum, significant difference was found between HC and PD patients, and for the connection from cerebellum to peripheral muscles significant difference was found between PD and PDC patients. No other significant differences were found in the other connections above.


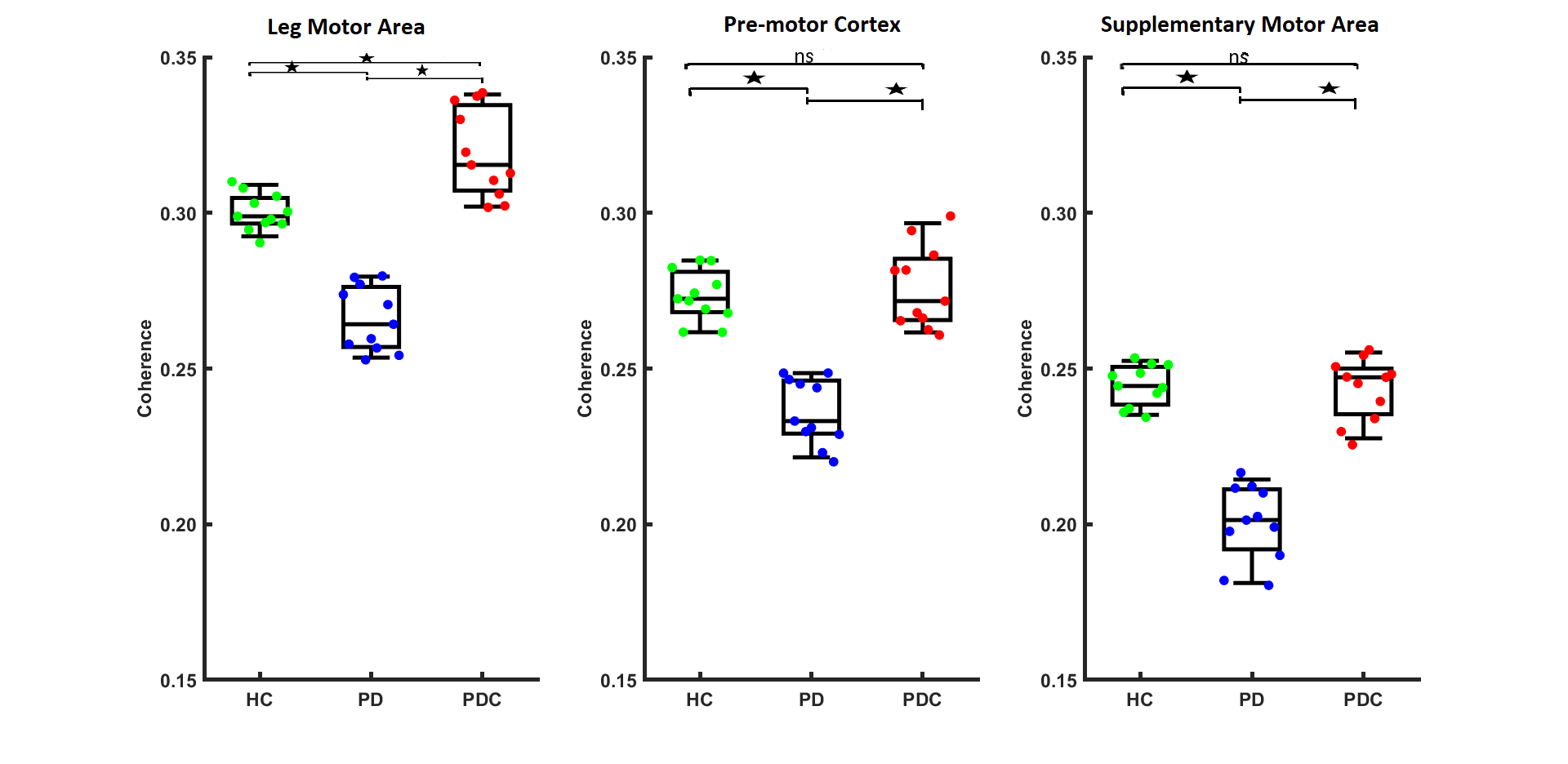


Figure S7: This figure shows the coherence values for the common coherent regions of the brain in the different groups of subjects Healthy controls, Parkinson’s patients without camptocormia (PD) and Parkinson’s patients with camptocormia (PDC). The stars at the top show a significant difference between the coherence values for two groups.


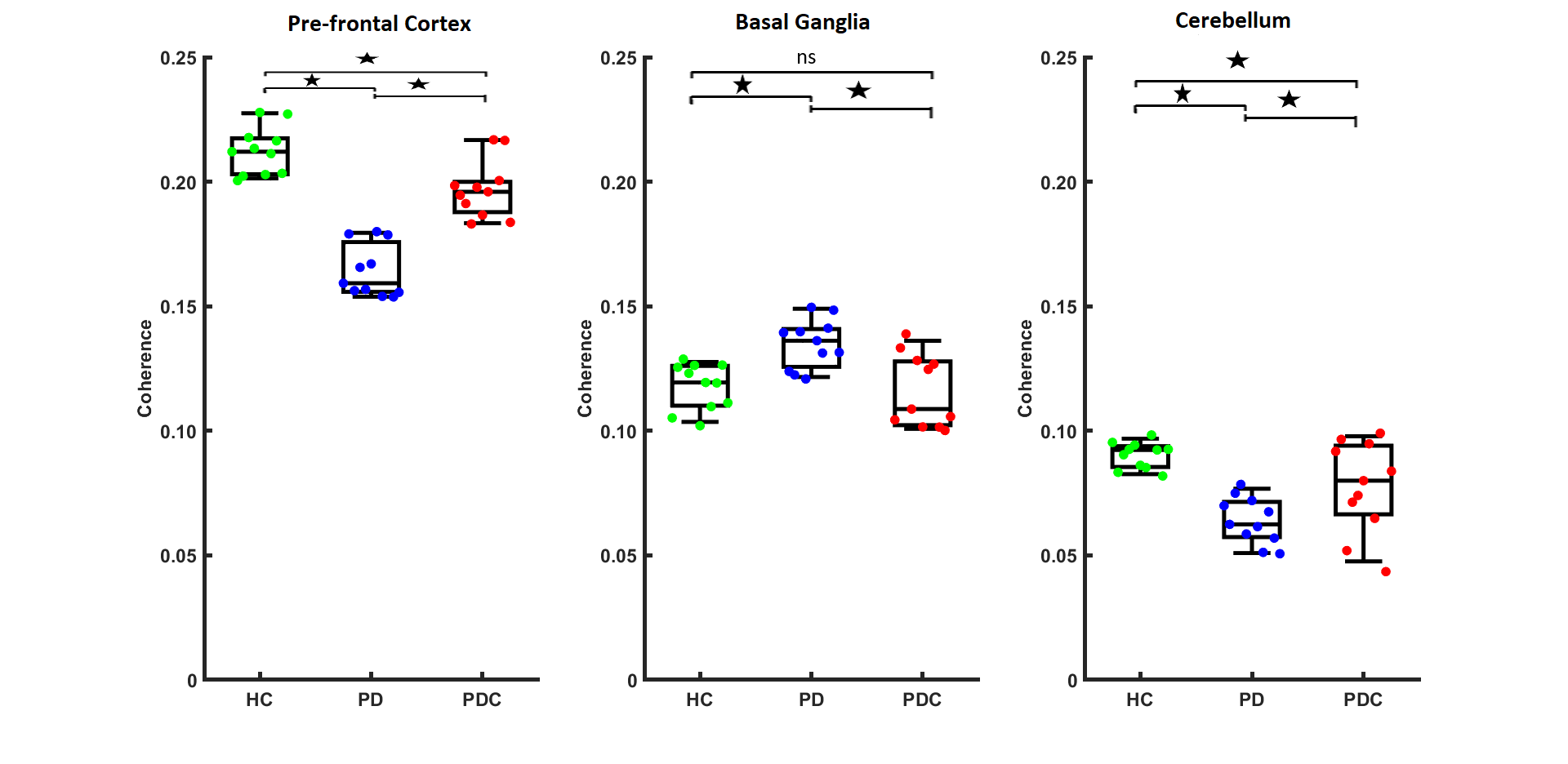


Figure S8: This figure shows the coherence values for the common coherent regions of the brain in the different groups of subjects Healthy controls, Parkinson’s patients without camptocormia (PD) and Parkinson’s patients with camptocormia (PDC). The stars at the top show a significant difference between the coherence values for two groups.


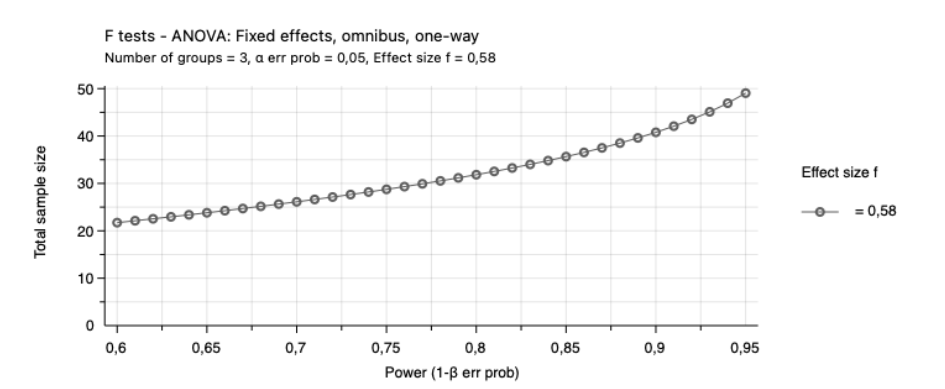


Figure S9: This figure shows a post-hoc power calculation using G-Power with an effect size of d = 0.58 based on the coherence difference and come to a total sample size of n=33 for the three groups.

Table S1: This table provides an overview of the patients included in the study, along with detailed information regarding their use of dopamine agonists, including the specific agents prescribed.

| Patient No. | Disease | Dopamine Agonist |
| --- | --- | --- |
| 1 | PD with Camptocormia | Rotigotine |
| 2 | PD with Camptocormia | Ropinirole |
| 3 | PD with Camptocormia | Pramipexole |
| 4 | PD with Camptocormia | Rotigotine |
| 5 | PD with Camptocormia | Pramipexole |
| 6 | PD with Camptocormia | None |
| 7 | PD with Camptocormia | None |
| 8 | PD with Camptocormia | Pramipexole |
| 9 | PD with Camptocormia | Rotigotine |
| 10 | PD with Camptocormia | Pramipexole |
| 11 | PD with Camptocormia | Rotigotine |
| 12 | PD without Camptocormia | None |
| 13 | PD without Camptocormia | Rotigotine |
| 14 | PD without Camptocormia | Not found |
| 15 | PD without Camptocormia | Rotigotine |
| 16 | PD without Camptocormia | Pramipexole |
| 17 | PD without Camptocormia | None |
| 18 | PD without Camptocormia | Ropinirole |
| 19 | PD without Camptocormia | Ropinirole |
| 20 | PD without Camptocormia | Ropinirole |
| 21 | PD without Camptocormia | None |
| 22 | PD without Camptocormia | Ropinirole |

**Supplementary References**

1. Welch P. The use of fast Fourier transform for the estimation of power spectra: a method based on time averaging over short, modified periodograms. *IEEE Trans Audio Electroacoustics*. 1967;15:70-73.

2. Halliday DM, Rosenberg JR, Amjad AM, Breeze P, Conway BA, Farmer SF. A framework for the analysis of mixed time series/point process data–theory and application to the study of physiological tremor, single motor unit discharges and electromyograms. *Prog Biophys Mol Biol*. 1995;64:237-78.

3. Muthuraman M, Galka A, Deuschl G, Heute U & Raethjen J. Dynamical correlation of non-stationary signals in time domain—A comparative study. Biomedical Signal Processing and Control. 2010;5(3):205-213.

4. Mitra PP, Pesaran B. Analysis of dynamic brain imaging data. . *Biophysical journal*. 1999;76(2):691-708.

5. Rosenberg JR, Amjad AM, Breeze P, Brillinger DR, Halliday DM. The Fourier approach to the identification of functional coupling between neuronal spike trains. . *Progress in biophysics and molecular biology,*. 1989;53(1):1-31.

6. Amjad AM, Halliday D M, Rosenberg JR & Conway BA. An extended difference of coherence test for comparing and combining several independent coherence estimates: theory and application to the study of motor units and physiological tremor. *Journal of neuroscience methods*. 1997;73:69-79.

7. Gross J, Kujala J, Hämäläinen M, Timmermann L, Schnitzler A & Salmelin R. Dynamic imaging of coherent sources: studying neural interactions in the human brain. *Proceedings of the National Academy of Sciences*. 2001;98(2):694-699.

8. Wolters CH, Anwander A, Berti G & Hartmann U. Geometry-adapted hexahedral meshes improve accuracy of finite-element-method-based EEG source analysis. *IEEE Transactions on Biomedical Engineering*. 2007;54(8):1446-1453.

9. Van Veen BD, Van Drongelen W, Yuchtman M, Suzuki A. Localization of brain electrical activity via linearly constrained minimum variance spatial filtering. *IEEE Transactions on biomedical engineering,*. 1997;44(9):867-880.

10. Muthuraman M, Raethjen J, Hellriegel H, Deuschl G & Heute U. Imaging coherent sources of tremor related EEG activity in patients with Parkinson's disease. IEEE; 2008:4716-4719.

11. Muthuraman M, Heute U, Deuschl G & Raethjen J. The central oscillatory network of essential tremor. IEEE; 2010:154-157.

12. Sekihara K, Scholz B. Generalized Wiener estimation of three-dimensional current distribution from biomagnetic measurements. *IEEE transactions on biomedical engineering*. 1996;43(3):281-291.

13. Gross J, Ioannides AA. Linear transformations of data space in MEG. *Physics in Medicine & Biology*. 1999;44(8):2081.

14. Gross J, Timmermann L, Kujala J, Dirks M, Schmitz F, Salmelin R & Schnitzler A. The neural basis of intermittent motor control in humans. *Proceedings of the National Academy of Sciences*. 2002;99(4):2299-2302.

15. Haykin S. *Kalman filtering and neural networks*. John Wiley & Sons; 2001.

16. Wan EA, Nelson AT. Dual extended Kalman filter methods. Kalman filtering and neural networks,. *Dual extended Kalman filter methods Kalman filtering and neural networks,*. 2001:123-173.

17. Kaminski M, Ding M, Truccolo WA & Bressler SL. Evaluating causal relations in neural systems: Granger causality, directed transfer function and statistical assessment of significance. *Biological cybernetics*. 2001;85:145-157.

18. Neumaier A, Schneider T. Estimation of parameters and eigenmodes of multivariate autoregressive models. *ACM Transactions on Mathematical Software (TOMS),*. 2001;27(1):27-57.

19. Schneider T, Neumaier A. Algorithm 808: ARfit—A Matlab package for the estimation of parameters and eigenmodes of multivariate autoregressive models. *ACM Transactions on Mathematical Software (TOMS)*. 2001;27(1):58-65.

20. Haufe S, Nikulin VV, Müller KR & Nolte G. A critical assessment of connectivity measures for EEG data: a simulation study. *Neuroimage*. 2013;64:120-133.
